# Polyinfection in Fish Aeromoniasis: A Study of Co-Isolated *Aeromonas* Species in *Aeromonas veronii* Outbreaks

**DOI:** 10.3390/pathogens12111337

**Published:** 2023-11-10

**Authors:** Yanelys Cantillo Villa, Adriana Triga, Pantelis Katharios

**Affiliations:** 1 Institute of Marine Biology, Biotechnology and Aquaculture (IMBBC), Hellenic Centre for Marine Research (HCMR), 71500 Gournes, Greece; ycantillovi@unal.edu.co (Y.C.V.); triga@hcmr.gr (A.T.); 2 Department of Biology, University of Crete, 71110 Heraklion, Greece; 3 Aquatic Biologicals, Thalassocosmos, 71500 Gournes, Greece

**Keywords:** antibiotic resistance, virulence factors, insertion sequences, European seabass, Mediterranean aquaculture, mesophilic *Aeromonas salmonicida*, *Aeromonas rivipollensis*

## Abstract

We studied the phenotypic and genomic characteristics related to the virulence and antibiotic resistance of two *Aeromonas* strains, which were co-isolated before an outbreak of *Aeromonas veronii* among diseased seabass on Agathonisi Island, Greece, in April 2015. The first strain, AG2.13.2, is a potentially pathogenic mesophilic variant of *Aeromonas salmonicida*, and the second, AG2.13.5, corresponds to an *Aeromonas rivipollensis* related to *A. rivipollensis* KN-Mc-11N1 with an ANI value of 97.32%. AG2.13.2 lacks the type III secretion system just like other mesophilic strains of *A. salmonicida*. This characteristic has been associated with lower virulence. However, the genome of AG2.13.2 contains other important virulence factors such as type II and type VI secretion systems, and toxins such as *rtxA*, aerolysin *aer*/*act*, and different types of hemolysins. The strain also carries several genes associated with antibiotic resistance such as the *tetE* efflux pump, and exhibits resistance to tetracycline, ampicillin, and oxolinic acid. In an in vivo challenge test with gilthead seabream larvae, the *A. veronii* bv *sobria* strain AG5.28.6 exhibited the highest virulence among all tested strains. Conversely, both *A. salmonicida* and *A. rivipollensis* showed minimal virulence when administered alone. Interestingly, when *A. veronii* bv *sobria* AG5.28.6 was co-administered with *A. rivipollensis*, the larvae survival probability increased compared to those exposed to *A. veronii* bv *sobria* AG5.28.6 alone. This finding indicates an antagonistic interaction between *A. veronii* bv *sobria* AG5.28.6 and *A. rivipollensis* AG2.13.5. The co-administration of *A. veronii* bv *sobria* AG5.28.6 with *Aeromonas salmonicida* did not yield distinct survival probabilities. Our results validate that the primary pathogen responsible for European seabass aeromoniasis is *Aeromonas veronii* bv *sobria*.

## 1. Introduction

Over the past ten years, *Aeromonas veronii* bv *sobria* has emerged as a significant pathogen within the Mediterranean aquaculture sector and it has been associated with considerable morbidity and mortality in the European seabass (*Dicentrarchus labrax*) which is one of the most important aquaculture species of the Mediterranean [1]. Different species of *Aeromonas* have been commonly isolated from the same events/screenings, with the most common isolated species being *Aeromonas media, Aeromonas salmonicida, Aeromonas bestiarum, Aeromonas hydrophila, Aeromonas caviae, Aeromonas molluscorum*, and *Aeromonas bivalvium* [2,3,4]. Moreover, there are multiple reports on cases of *Aeromonas* co-infections along with other bacteria, viruses, and parasites in fish such as tilapias (*Oreochromis* spp.), zebrafish (*Danio rerio*), striped mullet (*Mugil cephalus*), and golden trout *(Oncorhynchus chrysogaster)* among others [5,6,7,8]. The evidence suggests that in many cases, aeromoniasis can have a polymicrobial etiology.

Bacterial co-infections in fish are rather common and it has been shown that they amplify mortality rates and give rise to conditions such as ulcerative dermatitis and intestinal hemorrhage. The interactions between various pathogens can be synergistic, antagonistic, or neutral, triggering complex and varied immune responses [9]. In addition to the presence of different bacterial species, we must consider yet another complexity layer that arises from the advances in comparative genomics that have shown the significance of having also different bacterial strains. For example, regarding *Aeromonas*, an infection that would have been typically classified as monomicrobial is now considered a mixed infection in which four different strains of *Aeromonas hydrophila* are interacting with the host and in which the differences in the strains have been proven crucial in shaping the immune system response and therefore determining the outcome of the infection [10]. This newfound level of complexity challenges the paradigm of mono- vs. polymicrobial infections. The scenario we are confronted with is one where virulence is determined not solely by genetic content and strain characteristics but also by the intricate microbe-microbe and community–host interactions in the so-called “pathobiome era”. Those complex interactions require infection models that can assess possible synergistic, antagonistic, and neutral effects. For this purpose, the authors have proposed the use of organisms such as the fruit fly (*Drosophila melanogaster*), the worm (*Caenorhabditis elegans*), and the zebrafish (*Danio rerio*) [11,12,13]. In *C. elegans*, pairs of *Aeromonas* strains were shown to have enhanced virulence. The fruit fly model has also been recently used to assess the effects of polymicrobial infections with *Aeromonas* and has been proven successful [14].

Between April and September of 2015, during outbreaks of *Aeromonas* disease in European seabass occurring on the island of Agathonisi in Greece, different strains of *A. veronii* bv. *sobria* were isolated along with *A. salmonicida*, *Aeromonas rivipollensis*, and *A. bivalvium.* Even though *A. veronii* bv. *sobria* was identified as the etiological agent, it was reported that *A. salmonicida* and *A. rivipollensis* (erroneously recognized as *A. media*) were co-isolated from the same fish prior to infection with *A. veronii* bv. *sobria* [4]. This study aimed to assess the role of two *Aeromonas* co-isolates from diseased seabass during an outbreak of *Aeromonas veronii* bv. *sobria.* We characterized the strains *A. salmonicida* AG2.13.2 and *A. rivipollensis* AG2.13.5 both at the phenotypic and genomic levels. We assessed their virulence alone and in combination with other strains using a gilthead seabream (*Sparus aurata*) larva infection model.

## 2. Materials and Methods

### 2.1. Bacterial Strains

The bacterial strains that were used for experiments and comparisons are part of the bacterial collection of the Aquaculture Microbiology Laboratory of the Institute of Marine Biology, Biotechnology and Aquaculture (IMBBC) of the Hellenic Centre for Marine Research (HCMR) in Heraklion, Crete, which curates a large list of fully characterized bacterial pathogens. The names of the strains used and their NCBI genome accession numbers are as follows: *Aeromonas salmonicida* AG2.13.2 (JAIVBC000000000.1), *Aeromonas rivipollensis* AG2.13.5 (JAWHKQ000000000.1), and the *Aeromonas veronii* bv. *sobria* strains AG5.28.6 (NZ_NNSE00000000.1), BIOO050A (NZ_NPKD00000000.1), NS6.15.2 (NZ_NPKC00000000.1), NS (NZ_NMUR00000000.1), NS2 (NZ_NPKE00000000.1), NS13 (NZ_NQMB00000000.1), NS22 (NZ_NQMC00000000.1), PDB (NZ_NMUS00000000.1), and VCK1 (NZ_NNSF00000000.1) The *A. veronii* bv. *sobria* strains used for the experiments and comparisons were described in detail by Smyrli et al. (2019) [4]. Well-described and referenced whole-genome sequences of other *A. salmonicida, A. media,* and *A. rivipollensis* strains were retrieved from the NCBI to be used as references. The complete list of genomes can be found in Supplementary Table S1 for reference.

### 2.2. Genomic Analysis

The complete genomes of strains *A. salmonicida* AG2.13.2 and *A. rivipollensis* AG2.13.5 were sequenced utilizing paired-end technology (PE100) using a DNB seq platform (BGI Tech Solutions, Hong Kong) with a DNBSEQ-G400 sequencer. DNA was extracted from cultures grown overnight in TS broth at a temperature of 25 °C, employing the DNeasy Blood and Tissue kit (QIAGEN, Hilden, Germany). The library preparation process for the platform involved fragment selection, end repair, A-tailing, ligation of bubble adaptors, PCR amplification, splint circularization, digestion, purification, and creation of DNA nanoballs (DNBs). To ensure data quality, raw reads underwent rigorous filtering. Reads were excluded if they contained adapter sequences matching more than 25% of their length. Additionally, reads were removed if over 50% of their bases had quality values below 20 or if they contained more than 3% ambiguous bases (N). The filtering procedure was performed using SOAPnuke [15]. For the de novo assembly, we used the Unicycler genome assembler [16] of the Pathosystems Resource Integration Centre (PATRIC) [17]. Pilon software version 1.23 [18] was then used to polish the assembled contigs further to obtain high-quality genomes. The assembly was assessed by Benchmarking Universal Single-Copy Orthologs (BUSCO) [19,20] in the Galaxy platform [21].

The genomes of *A. salmonicida* AG2.13.2 and *A. rivipollensis* AG2.13.5 were annotated using the Prokka v.1.14.6 annotation pipeline [22] Rapid Annotation using the Subsystem Technology (RAST) server v.2.0 [23] and PATRIC annotation tool v.3.6.12 [17]. A visualization of the genome was built in the CG View Server [24]. Finally, genomes were submitted to the NCBI to be annotated through the PGAP annotation pipeline [25] under accession numbers (JAIVBC000000000.1) and (JAWHKQ000000000.1), respectively, along with their clean reads in SRA database. 

Genomic islands (GEIs) were predicted through the online platform Island Viewer v.3 [26] which includes three methods, IslandPick, IslandPath-DIMOB, and SIGI-HMM, for the prediction and detection of GIs. Virulence genes were predicted using the PATRIC annotation platform [17], which combines three databases: PATRIC_VF, VFDB [27], and VICTORS. Insertion sequences (ISs) were identified through the IS Finder web tool [28] and antibiotic resistance genes were predicted by the Resistance Gene Identifier (RGI) Software of the CARD platform [29]. To identify clustered regularly interspaced short palindromic repeats (CRISPRs), we used the CRISPRcasFinder tool [30]. Prophages were identified with the PHASTER phage search tool [31]. Genome similarity with other *Aeromonas* strains was assessed using Average Nucleotide Identity by Orthology with Ortho-ANI software [32] as part of the bacterial identification. 

### 2.3. Phenotypic Characterization

The strains of *A. salmonicida* AG2.13.2 and *A. rivipollensis* AG2.13.5 were cultured at 25, 30, and 37 °C using tryptic soy agar (TSA) to assess their mesophilic characteristics. Bacterial growth was measured using a spectrophotometer at OD 600 from 20 to 24 h post-incubation. 

The microscopical examination was conducted both with light and transmission electron microscopy (TEM). Special attention was given to the presence of motility structures. Bacteria were grown for 24 h hours in tryptic soy broth (TSB) and preserved in 2.5% formaldehyde in phosphate buffer. Bacteria preparations were negatively stained with 4% (*w*/*v*) uranyl acetate (pH 7.2) and observed with a JEOL JEM2100 (JEOL Ltd., Tokio, Japan) transmission electron microscope operated at 80 kV. TEM was conducted at the Electron Microscopy Laboratory of the University of Crete. 

The mass spectra of bacterial ribosomal cell proteins of *A. salmonicida* AG2.13.2 were obtained by MALDI-TOF MS using the VITEK MS system (bioMérieux SA, Marcy-l’Étoile, France) and a VITEK MS-CHCA matrix in the Department of Microbiology of the University Hospital of Heraklion. We compared the mass spectra acquired for the strain AG2.13.2 with those of the *A. salmonicida* reference strains reported by Fernández-Álvarez et al. (2016) [33] to assist bacterial identification. The sample was also compared to the known mass spectra in the database and given a confidence score according to how closely the acquired spectra matched those in the database.

Biochemical characterization was conducted with the commercial kit GEN III Microplate (BIOLOG, Hayward, CA, USA). Results were recorded after 48 h of incubation. Motility was also assessed on motility, indole, and ornithine (MIO) medium (Sigma-Aldrich, Co., St. Louis, MO, USA). Hemolytic activity was evaluated by visual inspection of colonies cultured in 5% gilthead seabream (*Sparus aurata*) blood agar plates after 24 h of incubation. The plates were incubated at 25 and 30 °C. Blood was collected aseptically from gilthead seabreams held at the facilities of the Institute, and blood agar plates were prepared using commercial blood agar base (Neogen Corporation, Lansing, MI, USA).

Antimicrobial susceptibility was assessed by the Kirby–Bauer disk diffusion method [34], using commercial 6 mm disks (Oxoid Ltd., Basingstoke, Hampshire, UK) on Muller–Hilton agar (Difco, Sparks, MD, USA). The inhibition diameter was recorded after 24 h incubation at 25 °C for the antibiotics oxytetracycline, tetracycline, piperacillin, sulfamethoxazole, florfenicol, ampicillin, flumequine, and oxolinic acid. 

### 2.4. In Vivo Virulence

Gilthead seabream larvae were used to assess the virulence of the strains *A. salmonicida* AG2.13.2, *A. rivipollensis* AG2.13.5, and *A. veronii* bv. *sobria* AG5.28.6. This method has been used and validated in the past at the Aquaculture Microbiology Lab of the IMBBC, HCMR, to assess the virulence of pathogenic bacteria as described by Droubogiannis et al. (2023) [35]. The gilthead seabream eggs for the experiment were collected from the HCMR hatchery on the same day. The fertilized eggs were carefully collected with a sieve in a 500 mL beaker with seawater and placed in an incubator at 19.6 °C. After some minutes in the incubator, the eggs were washed three times with previously filtered and sterilized seawater. The eggs were then placed individually in a 96-well microplate (1 egg/well) containing 200 μL sterile seawater. This was considered infection day 0. After day 0, the egg quality was evaluated daily every 24 h. The bacterial inoculation was performed after the hatching of the larvae by static immersion at day 1 and the survival of the larvae was monitored for the following 5 days. GraphPad Prism version 10.0.0 for Windows (GraphPad Software, San Diego, CA, USA) was used to construct Kaplan–Meier survival curves. The bacteria used in the challenge test were grown in TSA supplemented with 0.5% NaCl for 24 h at 25 °C and bacterial overnight cultures were used to prepare bacterial suspensions that were later used to inoculate the wells. The titer of the overnight cultures was calculated by serial dilution and following OD measurements that were correlated to CFU counts. Bacterial titer in the overnight cultures was calculated to be approximately 10^9^ CFU mL^−1^. Two mL of bacterial overnight culture was centrifuged and resuspended in saline to create a bacterial suspension. We used 20 µL of the bacterial suspension on each well containing 200 µL (20 μL of seawater was removed before the addition of the 20 μL of the bacterial suspension) of previously filtered and sterilized seawater to adjust the bacterial titer to be 10^8^ CFU mL^−1^ in each well, as suggested by Saraceni et al. (2016) [12]. When bacterial suspensions were prepared in combination, we used a proportion of 1:1.

## 3. Results and Discussion

### 3.1. Genomic Analysis

#### 3.1.1. Genome Assemblies and Characteristics

The statistics of the de novo assembly are shown in Supplementary Table S2 and the genomic features of the strains are summarized in Table 1. According to the statistics, both assemblies were considered of good quality with high coverage and N50s far above 50kb. According to the BUSCO analysis, the genome of *Aeromonas salmonicida* was assessed as complete, as shown in Supplementary Figure S1. On the other hand, the genome of *Aeromonas rivipollensis* is missing 2 BUSCO genes: the 50S ribosomal protein L18 and a large ribosomal RNA subunit accumulation protein encoded by the gene *YceD*.

Upon genome comparison, we observed a lower number of rRNAs and tRNAs in both our strains compared to the psychrophilic *A. salmonicida* reference strain A449 and the *Aeromonas rivipollensis* KN-Mc-11N1 reference strain (Table 1). A smaller number and low diversity of tRNAs have been associated with lower optimal growth temperatures and growth rates in archaea and other thermophilic bacteria. Having a high amount of tRNAs is also associated with higher efficiencies in protein translation [36,37]. It has also been suggested that psychrophilic strains of *Aeromonas* contain more tRNAs to compensate for the lack of motility [38]. From a scientific perspective, our results are in opposition to these assumptions as mesophilic strains often contain an equally high or even higher number of tRNAs than their psychrophilic counterparts as is the case with the mesophilic strain of *A. salmonicida* SRW-OG1. Given the amount of genomic information available for *Aeromonas*, it would be interesting to explore these assumptions further.

#### 3.1.2. Genome Similarity

**Figure 1:**
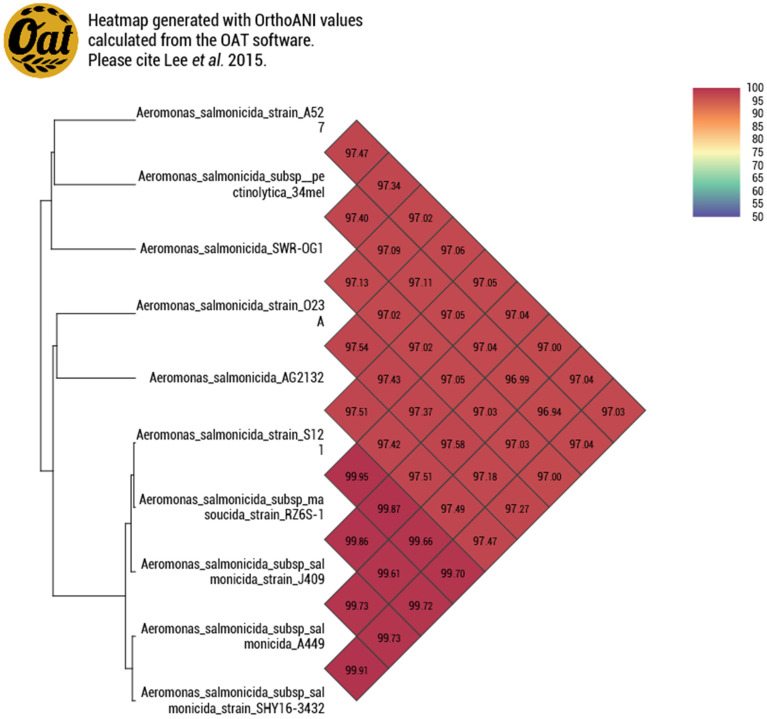
Genome similarity heatmap with OrthoANI values for *A. salmonicida* AG2.13.2 compared to other *A. salmonicida* retrieved from the NCBI dataset.

These results are evidence of the diversity of *A. salmonicida* and corroborate the fact that it is possible to find mesophilic and psychrophilic strains in the same place [39]. The European seabass is the perfect example since it is a eurythermal species and there are reports of infection caused by both psychrophilic and mesophilic strains of *A. salmonicida* [40]. Vincent et al. (2017) [41] suggested a clustering of two distinct groups of *A. salmonicida*: the first containing the psychrophilic subspecies *masoucida* and *salmonicida*, and the second containing the mesophilic *A. salmonicida* strains A527, Y47, Y567, and Y577 and the mesophilic subspecies *pectinolytica*. This classification was corroborated by the Average Nucleotide Identity of AG2.13.2 (Figure 1). We used nine reference strains to build the similarity heatmap and we can observe that the strains of the subspecies *salmonicida* clustered together with the *masoucida* strain RZ6S-1 and the strains A527 and SRW-OG1 clustered with the *pectinolytic* 34mel strain as predicted by the literature [38]. On the other hand, strain AG2.13.2 clustered with strain O23A. According to Uhrynowski et al. (2017) [42], the strain O23A is related to *A. salmonicida* subsp. *salmonicida* A449 and exhibits characteristics such as tolerance to changes in pH (4–11) and temperature (4–42 °C) and it was found to be capable of reducing arsenic. Only two *Aeromonas* spp. strains have been reported to be dissimilatory arsenate reductase positive. The source of both these strains is the Zloty Stok gold main (SW Poland). These strains correspond to O23A and OM4 which are unclassified [43,44].

**Figure 2:**
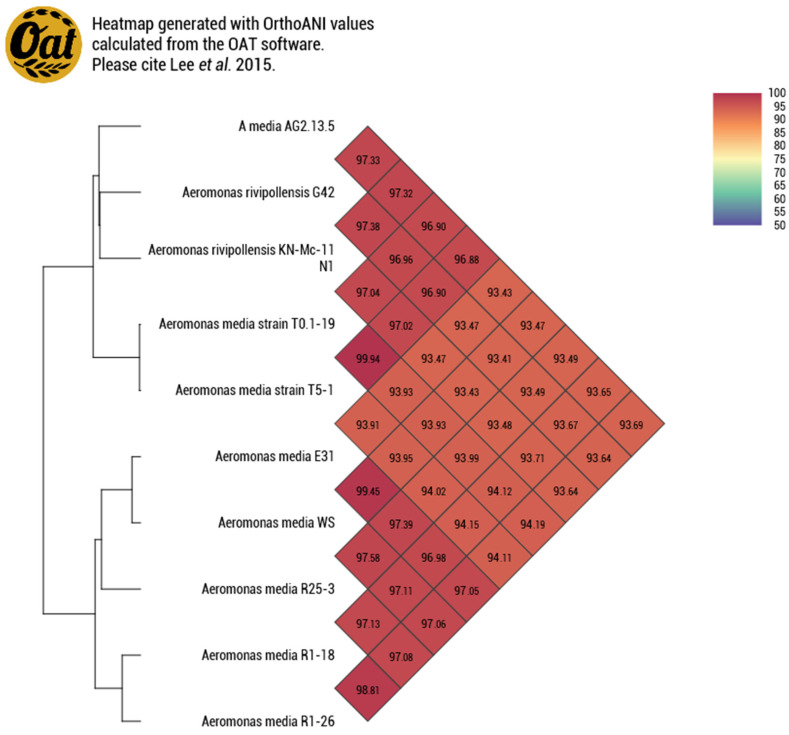
Genome similarity heatmap with OrthoANI values for the *A. rivipollensis* AG2.13.5 genome compared with other genomes of *A. media* retrieved from the NCBI dataset.

After the whole genome analysis, the Average Nucleotide Identity revealed that the strain AG2.13.5 clustered with two Chinese isolates from a biofilm in a bioreactor containing oxytetracycline wastewater [45]. These isolates were misidentified as *A. media* and correspond to the strains T0.1-19 and T5-1. The genome of the strain T5-1 has been described by Shi et al. (2021) [46]; meanwhile, information regarding the strain T0.1-19 remains unpublished. Nevertheless, according to the ANI results, the three genomes AG2.13.5, T0.1-19, and T5-1 clustered with *A. rivipollensis* strains KN-Mc-11N1 and G42 with an ANI value of 97.32%, suggesting a relationship (Figure 2). A commonly used guideline to differentiate between bacterial species is to consider an ANI threshold of 95–96%. When the ANI value between two genomes falls below this threshold, it suggests that they are likely from distinct species. 

#### 3.1.3. Prediction of Genomic Islands, CRISPR Systems, Prophages, and Insertion Sequences

A total of 42 genomic islands (GEIs) were predicted in *A. salmonicida* AG2.13.2 with an average length of 11,991.7 bp and a total length of 503,650 bp. These represent around 10.5% of the genome. We have enumerated the islands from 1 to 42 and registered their start and end base pair positions. Islands 1, 2, 12, and 13 are particularly interesting. GEI1 contains genes related to heavy metal resistance, including *cusA* (LCG94_19930) and *cusB* (LCG94_00450), which are part of a cation efflux system that mediates resistance to copper and silver. It also contains genes for a lead, cadmium, zinc, and mercury-transporting ATPase (LCG94_21615) and a copper-translocating P-type ATPase (LCG94_01075). GEI2 contains, among other genes, an outer membrane outer membrane protein OmpA (LCG94_12850) and a phage immunity repressor *protein C* (LCG94_09700). GEI12 has, among others, a plasmid replication initiation protein, multiple conjugative transfer proteins, and a plasmid stabilization protein. The tetracycline resistance regulatory protein TetR (LCG94_08205) and the tetracycline resistance efflux pump TetE (LCG94_08210) are found in three different islands including the GEI13. Islands GEI17, 18, and 19 contain a small multidrug resistance family (*SMR*) protein (LCG94_08330). GEI39 and GEI41 both encode transposase *insH* (LCG94_00175 and LCG94_07275) for the insertion sequence element IS5. Both islands encode mostly hypothetical proteins but also some genes such as *umuD* (LCG94_00205), *umuC* (LCG94_00210), and *xre* (LCG94_22030) which are involved in the SOS response [47]. *Xre* is necessary for maintaining the lysogenic state of a prophage in *Bacillus subtilis* [48].

Ten genomic islands were identified in the AG2.13.5 genome, with a total of 140,586 base pairs and constituting 3.15% of the genome. Most of these islands contain genes encoding hypothetical proteins. It is noteworthy that genes within the identified Genomic Islands (GEIs) of AG2.13.5 include *umuD* (RXZ09_04310), *umuC* (RXZ09_04305), and the gene encoding protein YffB (RXZ09_04520), which belongs to the ArsC family. Additionally, the genomic islands harbor genes such as flagellin *hag3*, flagellar proteins *fliD* (RXZ09_05330), and *fliS* (RXZ09_05335), as well as the nitric oxide reductase transcription regulator norR (RXZ09_09275). The predictions also identified TolC (RXZ09_15840), MdtL family multidrug efflux MFS transporter (RXZ09_03810), and several copper resistance genes, including *copA3* (RXZ09_11845), *copB* (RXZ09_09335), and *cutC* (RXZ09_12885).

The CRISPR system is capable of identifying and silencing intruding functional elements. CRISPR sites are fragments of foreign DNA (spacers), separated by short palindromic repeats and grouped into clusters in intergenic regions. The CRISPR sites work as a record of potentially dangerous genetic information. The CRISPRCasFinder tool showed that AG2.13.2 has 1 CRISPR site with evidence level 2 and 13 sites with evidence level 1 (Supplementary Table S3). However, evidence level 2 CRISPR sites likely correspond to invalid CRISPR arrays [30]. The PHASTER phage search tool showed the presence of one incomplete prophage in the genome of *A. salmonicida* AG2.13.2, the details of which are shown in Supplementary Table S4. No CRISPR sites or phages were found in the genome of *A. rivipollensis* AG2.13.5. 

A total of 23 ISs were identified in the genome of AG2.13.2; 13 were complete, 5 were incomplete, and 8 were considered uncategorized according to the IS finder results [28]. The IS finder predicted that *A. salmonicida* AG2.13.2 and the mesophilic strains pectinolytica, Y577, Y567, Y47, A527, and SRW-OG1 contain the IS3 family ISAs31 in different copy numbers. However, this IS was not found in the O23A strain. The mesophilic strains AG2.13.2, A527, and SRW-OG1 have, in almost the same region of their genomes, ISChy3 which belongs to the IS481 family and an IS belonging to the IS1595 family. For AG2.13.2, O23A, and SRW-OG1, the IS of the IS1595 family is ISSsu9. In strain A527, they are ISKpn3 and ISAs23. In comparison to the other strains used for this analysis, the A527 has the highest number of IS. This could be associated with a higher number of events of horizontal gene transfer or genomic rearrangements.

The genome of *A. rivipollensis* AG2.13.5 is predicted to contain ISChy3 and ISAeme21 which belong to the IS481 family as well as ISAeme10, a member of the IS1595 family. The isolates T0.1-19 and T5-1 contain multiple copies of ISAs7 which is one of the most common IS in the psychrophilic isolates of *A. salmonicida* [49]. According to Siguier et al. (2015) [50], the IS481 family mentioned above is recognized as a transpositionally active family that has played a key role in the genome evolution of *Bordetellae pertussis*, having undergone amplification and subsequent genome decay. 

**Figure 3:**
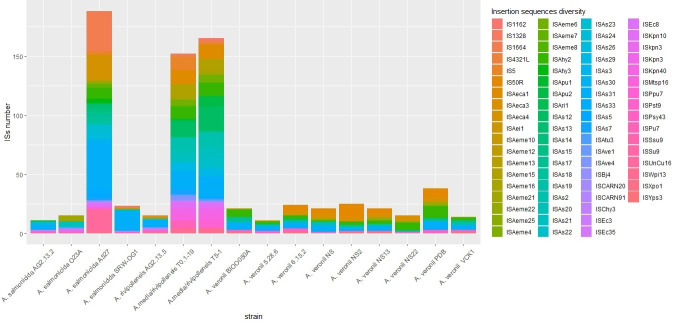
Comparison of insertion sequences encoded in the chromosomes of different strains of *A. salmonicida*, *A. rivipollensis*, and *A. veronii*.

Given its importance in the lifestyle changes of *A. salmonicida* [38] in adaptation to its environment and association with antibiotic resistance genes [46], we compared the distribution of complete ISs on the genomes of the strains from the incident and some of the reference strains retrieved from the NCBI (Figure 3). The details on the distribution of ISs in *A. salmonicida* have been further explored by Long et al. (2023) [49]. Overall, we noticed that the environmental isolate *A. salmonicida* A527 and the *A. media*/*rivipollensis* isolates under oxytetracycline stress T0.1-19 and T5-1 have a higher number of IS elements (see Figure 3). Despite some elements being unique to some strains or groups of strains, we identified common elements, such as ISAs31, that are present in different copy numbers in all the strains that we studied except the strain O23A. In fact, ISAs31 was the most common element with a high copy number in all the strains. This IS is present in all the mesophilic *A salmonicida* strains including subsp. *pectinolytica* [49]. Elements like ISSsu9, ISAs19, ISAChy3, and ISAhy3 are also widely distributed. The element ISPpu7 was only found in the oxytetracycline-resistant strains AG2.13.2, T0.1-19, and T5-1. Strains containing the same IS elements is an important indicator of a higher chance of horizontal gene transfer (HGT) between bacteria.

Horizontal gene transfer (HGT) plays a vital role in bacterial evolution and adaptation, enabling the exchange of DNA containing crucial genes, particularly those responsible for antibiotic and heavy metal resistance, as well as pathogenicity. These mechanisms encompass transformation, involving the direct absorption and integration of DNA by competent bacteria, with variable efficiencies in *Aeromonas*. Transduction, mediated by bacteriophages, has limited evidence of a significant role in genetic material transfer in *Aeromonas* and the most recognized mechanism within the genus is conjugation, which significantly contributes to antibiotic resistance dissemination within *Aeromonas* populations [51].

There is evidence demonstrating the successful acquisition of genetic material by conjugation in *Aeromonas* bacteria, even between phylogenetically distant species like *Vibrio cholerae*, *V. parahaemolyticus*, and *Yersinia ruckeri*, which serve as recipients of plasmids originating from *A. salmonicida* [52]. However, the mechanisms of pathogenicity in *Aeromonas* spp. remain poorly understood, as noted by authors like Piotrowska and Popowska (2015) [53]. They have also introduced the concept of the “mobilome” comprising all genetic elements, including integrons, transposons, conjugative or integrative elements, plasmids, and phages, that participate in horizontal transfer events.

#### 3.1.4. Antibiotic Resistance Genes

The genes related to antibiotic resistance are shown in Table 2. Due to the natural occurrence of phenotypes of β-lactamases producers, *Aeromonas* isolates have been reported to have a relatively high resistance to β-lactamic antibiotics. For example, ampicillin resistance of *Aeromonas* isolates associated with waste has been reported on multiple occasions [54,55,56,57]. The resistance to tetracycline and oxytetracycline of the strain AG2.13.2 can be explained by the presence of the usually plasmid-encoded genes *TetE* (LCG94_08210) that encodes for an efflux pump and *tetR* (LCG94_08205) which works as a regulator of TetE. Efflux proteins work by exchanging a proton (H^+^) for the tetracycline molecule against a concentration gradient, and therefore pumping the drugs out of the cell [58]. Additionally, AG2.13.2 also exhibits resistance to oxolinic acid. AG2.13.2 and AG2.13.5 both contain the plasmid-mediated quinolone resistance gene *qnrB* that protects bacterial topoisomerases (DNA gyrase and topoisomerase IV) from the activity of quinolones [59]. In particular, in *A. salmonicida* the *qnrB* is associated with mid- to low-level quinolone resistance [60]. A study by Barnes et al. (1990) [61] reported that cross-resistance to oxytetracycline and oxolinic acid was associated with the presence of a 37kDa outer membrane protein. On the other hand, *tetE* and *tetR* are absent in *A. rivipollensis* AG2.13.5.

#### 3.1.5. Virulence Factors

**Figure 4:**
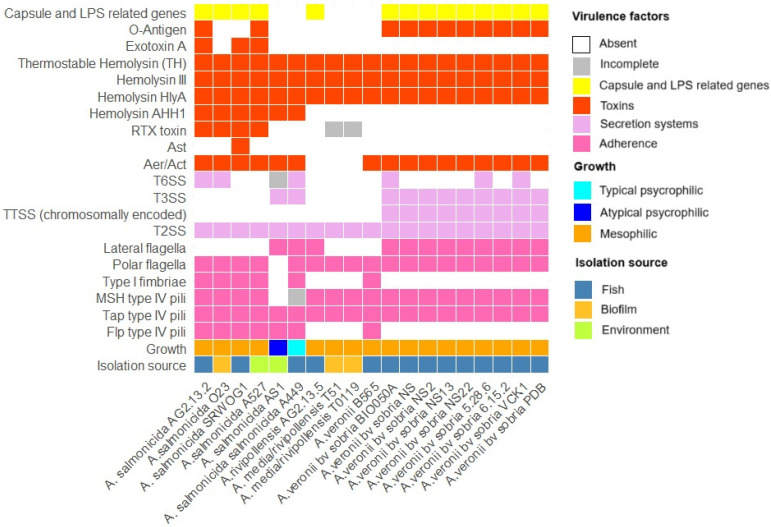
Comparison of virulence factors encoded in the chromosomes of different strains of *A. salmonicida*, *A. rivipollensis*, and *A. veronii.* The T3SS of A449 and AS are shown as present but both are encoded in plasmids.

The virulence factors identified for *A. salmonicida* AG2.13.2 and *A. rivipollensis* AG2.13.5 are shown in Figure 4. We compared these two strains with the *Aeromonas veronii* isolates from the incident reported by Smyrli et al. (2019) [4] and other *Aeromonas* reference strains from the NCBI. All the virulence genes and their accession numbers as predicted by the VFDB can be found in Supplementary Table S5. It is important to note that we only considered virulence factors encoded in the chromosomes, as no plasmids have been isolated from the strains of the *A. veronii* bv *sobria* incidents. However, it is known that the type III secretion system (T3SS) of *A. salmonicida* A449 and AS1 are encoded in plasmids, so for the purpose of this comparison, the T3SS of A449 and AS1 is marked as present in Figure 4.

The most important difference between *A. salmonicida* and *A. rivipollensis* when compared to the *A. veronii* strains bv. *sobria* from the incidents is the lack of a T3SS. All the *A. veronii* bv. *sobria* strains have two clusters of genes related to the T3SS. One cluster is similar to the one encoded in the plasmid pAsa5 (NCˍ009350) of *A. salmonicida* subsp. *salmonicida* A449 that contains the genes *acr12GHRV*, *aopBDN*, *ascBCDEFGHIJKLNOPQRSTUVXY*, *exsABCDE*, and *sycHOX* and the other cluster is related to the chromosomally encoded T3SS genes of *Yersinia: escC*/*yscC*/*hrcC*, *escV*/*yscV*/*hrcV*, *escN*/*yscN*/*hrcN*, *sctR*, *escS*/*yscS*/*hrcS*, and *escT*/*yscT*/*hrcT* [4]. 

The T3SS in *A. salmonicida* is described in Burr et al. (2002) [62] and its importance for virulence is well known [63]. The T3SS is used to inject specific effector proteins into the cytoplasm of the host cells, disrupting the cytoskeleton and inducing apoptosis [64]. The mechanism of loss of the T3SS of *A. salmonicida* is well explained by Dallaire-Dufresne et al. (2014) [65]; it is caused by an IS-mediated rearrangement that cuts out the T3SS locus from the pAsa5 plasmid through homologous recombination between two flanking copies of a specific IS (*ISAs11*) without deleting the rest of the plasmid [66]. Strains that are routinely grown at 25 °C have more complex patterns, which may have resulted from multiple, sequential rearrangements involving other ISs. Moreover, when the bacterium is cultivated at higher temperatures (25 °C), non-virulent mutants are produced [67,68].

The type II secretion system (T2SS) gene clusters were predicted to be encoded in all the strains. This system is known as the general secretory pathway and is used to transport aerolysin, amylases, DNases, and proteases [69]. The type VI secretion system (T6SS) gene clusters are present in *A. salmonicida* AG2.13.2 and in the *A. veronii* bv. *sobria* strains (BIO050A, 5.28.6, and VCK1). It is known that the T6SS locus of the psychrophilic *A. salmonicida* A449 is dysfunctional due to insertions and deletions [70]. Nevertheless, we have no information on the functionality of the T6SS of our *A. salmonicida* AG2.13.2.

*A. salmonicida* virulence has been typically associated with the presence of T3SSs. However, this does not seem to be the case for all *Aeromonas* species. For example, in *A. hydrophila*, virulence has been associated with the presence of T6SSs. According to Tekedar et al. (2019) [71], a study of 55 isolates of *A. hydrophila* from the U.S.A and China showed that virulent *A. hydrophila* isolates did not have a T3SS, but their virulence was greatly reduced whenever there was a deletion in the T6SS genes *hcp1* and *vgrG1*. This suggests that different secretion systems may be contributing differently to the virulence of different species.

*Aeromonas* species have two types of adhesins, filamentous (the flagella and fimbriae) and non-filamentous (LPS, capsule, and OMPs) [64]. The polar and lateral flagella genes are both present in the reference strain *A. salmonicida* A449 which is described as a psychrophilic non-motile strain. Deletions and ISs in the genes implicated in the formation of these adhesins in A449 are responsible for the incorrect expression of proteins and therefore the lack of motility [70]. On the other hand, lateral flagella genes were absent in the mesophilic *A. salmonicida* strains AG2.13.2, O23A, SRW-OG1, and A527 as suggested by Merino et al. (2003) [72]. Lateral flagella genes were predicted in *A. rivipollensis* AG2.13.5 but not in the Chinese isolates T0.1-19 and T5-1. Also, they were predicted in all the *A. veronii* bv. *sobria* strains from the incident. The genes of the polar flagella of *A. salmonicida* are similar to those of the sodium-driven flagellar systems described for *Vibrio alginolyticus*, *V. parahaemolyticus*, and *A. hydrophila* in which the polar flagellum stator complex is integrated by two essential proteins, MotX and MotY, which interact with one of two redundant pairs of proteins, PomAB and PomA2B2 [73], as predicted by the VFBD database. 

It is known that the genus *Aeromonas* is divided based on motility, which is dependent on two types of flagella: (a) polar unsheathed flagella, which are expressed in liquid environments, and (b) peritrichous lateral flagella, which are important in media where polar flagella are unable to propel the cell [72]. The polar flagellum has been demonstrated to be important for the adherence to and invasion of human and fish cell lines in mesophilic aeromonads [73]. Interestingly, it was reported by McIntosh and Austin (1991) [74] that supra-optimal incubation temperatures (30 to 37 °C) resulted in *A. salmonicida* isolates producing a polar sheathed flagellum. This also occurred when the isolates were grown in 18% (*w*/*vl*) Ficoll. However, whenever this occurred, it was always in less than 1% of the population.

Fimbriae or pili can be polar or lateral and help to enhance the adherence of the bacteria to solid surfaces or to host tissues. Among the four different types of pili (I-IV), type IV is the one most associated with virulence and it is associated with epithelial adherence, colonization, cellular invasion, and formation of biofilms. Nevertheless, *A. salmonicida* mutant strains for the type I fimbriae pili are less adherent to the gastrointestinal tract of Atlantic salmon [64].

The *A. salmonicida* strains have both type I and type IV pili. Meanwhile, all the *A. rivipollensis* and all the *A. veronii* bv. *sobria* strains from the incident lack the type I fimbria genes. Three clusters of type IV pili have been described in the literature: Tap, Flp, and Msh. While some *tap* genes are essential for the viability of the bacteria and stimulation of the fish immune response, the *flp* does not seem to have much relevance [64]. In fact, all the *A. veronii* bv. *sobria* strains of the incident that were determined as the causative agent of the disease outbreak and *A. rivipollensis* AG2.13.5 lacks the *flp* locus. Finally, the set of *msh* type IV pili genes is incomplete in the *A. salmonicida* psychrophilic strain A449 and absent in AS1, but complete in all the mesophilic *A. salmonicida*, *A. rivipollensis*, and *A. veronii* bv. *sobria* strains including the *A. salmonicida* AG2.13.2 and *A. rivipollensis* AG2.13.5 strains of this study.

It has been shown that mutations in pilin proteins of the *msh* pili in *A. veronii* bv. *sobria* reduced the bacterium’s ability to adhere to a human cell line and to form biofilms [75]. The complete locus of *msh* type IV pili is a feature that has been recently reported to be unique for the mesophilic group [49].

Capsule-related genes were predicted in all the strains except the psychrophilic *A. salmonicida* isolates A449 and AS1. The most predicted gene for all the strains was the *pseC* that encodes the enzyme UDP-4-amino-4,6-dideoxy-N-acetyl-beta-L-altrosamine transaminase which is involved in the synthesis and modification of nucleotide sugars. Other predicted genes related to capsule biosynthesis with their corresponding locus tags are shown in Supplementary Table S5. There are several studies that showed how bacteria possessing capsules have increased adhesion and invasion capacity against fish cell lines compared to bacteria without capsules, thus contributing to their virulence. For example, the capsule of *A. hydrophila* was found to provide resistance against the serum of the tilapia, *Oreochromis aureus*, and the phagocytic capacity of macrophages of the blue gourami, *Trichogaster trichopterus* [76,77].

LPSs have three domains: the O-polysaccharide, which triggers the production of specific antibodies; the core oligosaccharide; and the lipid A, which is attached to the bacterial membrane [78]. The LPS gene *kdsA* was predicted only in the *A. veronii* bv. *sobria* strains from the incident, AG5.28.6, BIOO050A, NS6.15.2, NS, NS2, NS13, NS22, PDB, and VCK1. The gene encodes the 3-deoxy-8-phosphooctulonate synthase which plays a pivotal role in the biosynthesis of a molecule known as 3-deoxy-D-manno-octulosonate-8-phosphate (KDO-8-P) in bacteria. KDO-8-P serves as a significant intermediate in the synthetic pathway of lipopolysaccharides (LPSs) within Gram-negative bacteria [79]. Genes related to the O-antigen were predicted only in the strains AG2.13.2, A527, T0.1-19, and T5-1. The most commonly predicted gene *ManB* encodes mannose-1-phosphate guanylyltransferase which facilitates the synthesis of guanosine diphosphate mannose (GDP-mannose). GDP-mannose is of paramount importance in multiple metabolic pathways, notably in the synthesis of lipoglycans. It acts as a precursor for the incorporation of mannose residues into glycoproteins and glycolipids [80].

Wang et al. (2007) [81] found that the O-antigen of typical and atypical *A. salmonicida* strains associated with fish infections can provoke diverse immune responses. This indicates that there are variations in the O-antigen composition and structure. Also, the core oligosaccharide has shown to be structurally unique for each subspecies of *A. salmonicida* [78].

An important virulence factor of *A. salmonicida* strains, which is associated with the capsule and LPSs, is the A-layer. It is a two-dimensional (2D) crystalline array that coats the entire cell, and it is thought to provide important functional properties as it serves as the interface between the cell and the environment. The structural gene for the A-layer of *A. salmonicida* is the gene *vapA* which encodes protein A [82]. It has been proposed that the O-polysaccharide side chains of LPSs are involved in anchoring the A-layer to the cell by an interaction mediated by divalent Ca^2+^ cations. Novel A-layer patterns were formed as a result of growth under conditions of calcium limitation and chelation of divalent cations with EDTA or EGTA. In such conditions, the A-protein was sometimes released as tetrameric units rather than in a monomeric form, forming distinct regular arrays according to Garduño et al. (1992) [83]. In this study, no *vapA* gene was found in the genomes of the mesophilic strains AG2.13.2, A527, or SRW-OG1, and Paquet et al. (2022) [84] reported that the Indian isolates (Y47, Y567, Y577) were also A-layer-negative. Several authors have shown that mesophilic strains of *A. salmonicida* lack the A-layer. According to Ishiguro et al. (1981) [85], *A. salmonicida* A450-1 loses the A-layer when cultured at high temperatures. These attenuated strains also happened to be phage-sensitive. A study about the A-layer of *A. salmonicida* subsp. *salmonicida* by Paquet et al. (2019) [86] reported how phage plaques were turbid or clear depending on whether the A-layer surface array protein was expressed or not. A genome analysis later revealed that the genes involved in the bacteriophage insensitivity of *A. salmonicida* subsp. *salmonicida* were more related to the biogenesis of lipopolysaccharides (LPSs) which were characterized as phage receptors rather than the absence of an A-layer. However, it is suggested that the A-layer can contribute to protection against phages to some degree by means of covering the exposed portion of LPSs.

Among the genes associated with hemolytic activity in *Aeromonas*, we predicted a thermostable hemolysin TH, hemolysin III, and *hlyA* to be encoded in all the strains analyzed in this study. We also predicted an Aerolysin *aerA*/*act* gene in all the strains analyzed except the *A. rivipollensis* strains AG2.13.5, T0.1-19, and T5-1. Both hemolysin and aerolysin are pore-forming toxins that are important for pathogenicity in *Aeromonas* [87]. Aerolysin is known for having both cytotoxic and enterotoxic activity. According to Chen et al. (2022) [87], the mesophilic lifestyle of *A salmonicida* could lead to the enhancement of hemolytic activity and other virulence factors. These authors showed that hemolytic activity was elevated at higher temperatures and the genes for *aerA* and *hlyA* were upregulated in the strain SRW-OG1. The extracellular hemolysin *Ahh1* gene is missing in the *A. rivipollensis* strains AG2.13.5, T0.1-19, and T5-1, and in all the *A. veronii* bv. *sobria* strains. The RTX toxins locus *rtxABCDEH*, and particularly *rtxA*, has been implicated in inducing host cell apoptosis [88]; the complete locus was only predicted in the mesophilic *A. salmonicida* strains. However, both the *A. rivipollensis* strains T0.1-19 and T5-1 are predicted to encode the *rtxA* gene. An exotoxin homolog of the exotoxin A (*exoA)* of *Pseudomonas aeruginosa* was predicted in the strains AG2.13.2, SRW-OG1, and A527. Exotoxin A is known to inhibit protein synthesis of host cells by covalently binding to elongation factor 2 and thus affecting polypeptide chain elongation [89].

### 3.2. Phenotypic Characterization

#### 3.2.1. Morphology and Biochemical Characteristics

**Figure 5:**
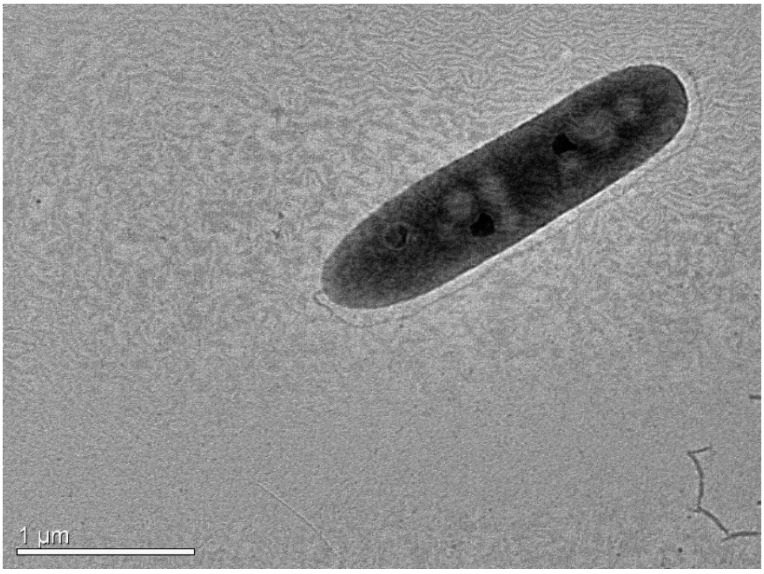
TEM micrograph of *A. salmonicida* AG2.13.2. The presence of a single polar flagellum is easily distinguished.

AG2.13.2 was first identified as *A. salmonicida* based on 16S rRNA and gyrB sequencing. The identification was further confirmed by a whole-genome analysis and biochemical characterization. Microscopic examination revealed the presence of a single polar flagellum and bacterial growth at 37 °C confirmed that the strain of *A. salmonicida* AG2.13.2 belongs to the mesophilic group (Figure 5).

It was reported by Fernández-Álvarez et al. (2016) [33] that MALDI-TOF MS analysis was able to accurately identify bacterial strains isolated from European seabass cultured in Spain as *A. salmonicida* subsp. *salmonicida* and this method could possibly differentiate between subspecies. The bacterial isolates from this study showed characteristic peaks and specific peak masses that allowed the discrimination of strains of *A. salmonicida* subsp. *salmonicida* from reference strains of different subspecies, so we decided to compare the mass spectra of AG2.13.2 with the ones in that study (Table 3).

The mass spectra reported by Fernández-Álvarez et al. (2016) [33] was generated by a Microflex MALDI-TOF-MS mass spectrometer (Bruker Daltonics GmbH & Co. KG, Bremen, Germany) equipped with a 337nm N_2_. From comparing the results, we could clearly identify characteristic peaks for all the strains of *A. salmonicida* (for example, the peaks 2028, 3595, 5156, and 7333). However, no subspecies-specific peaks matched the profile of AG2.13.2. Therefore, we can conclude that *A. salmonicida* AG2.13.2 likely does not belong to any of the known subspecies. According to Bergey’s manual (2007), five subspecies of *A. salmonicida* are recognized as psychrophilic and non-motile, and are considered “typical”. The atypical mesophilic strains show greater diversity and have proven difficult to differentiate. Overall, the phenotypic classification of atypical strains has been ambiguous [90]. We suggest that, as more mesophilic strains are described, comparing their profiles could be of interest.

Regarding the biochemical profile, the mesophilic strains of *A. salmonicida* share some signature traits such as the production of acid from sorbitol and arabinose, and the presence of active L-lysine decarboxylase and β-galactosidase. Additionally, they can be either cytochrome c oxidase-negative or positive. On the other hand, psychrophilic strains are only cytochrome-c-oxidase positive. AG2.13.2 is motile, oxidase- and citric acid-positive, ornithine- and indole-negative with no pigment production and contains the genes for L-lysine decarboxylase *cadA* (LCG94_19095) and β-galactosidase (LCG94_09560, LCG94_08610). Its GEN III Microplate biochemical profile corresponds to the *A. media*-like group A5 (*p* = 61.7%) (Table 4).

**Figure 6:**
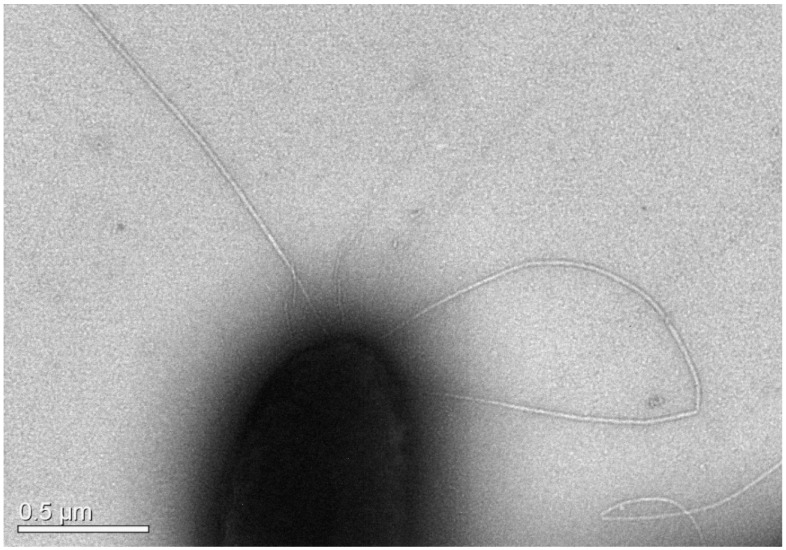
TEM micrograph of *A. rivipollensis* AG2.13.5 showing the presence of lophotrichous flagella.

The strain AG2.13.5 was first misidentified as *A. media* based on 16S rRNA and *gyrB* sequencing, but after a whole-genome analysis, it was identified as *A. rivipollensis.* According to the GEN III Microplate results, the biochemical profile of *A. rivipollensis* corresponds to the *A. media*-like group A5 (*p* = 74.8%). According to Bergey’s manual (2007), *Aeromonas media* is typically non-motile. Some of its biochemical characteristics include being ornithine decarboxylase-negative, lysine decarboxylase-negative, indole-positive, and oxidase-positive. *A. rivipollensis* AG2.13.5 is motile (Figure 6), indole-negative, ornithine decarboxylase-negative, and citric acid-negative (Table 4).

One of the metabolic differences between *A. salmonicida* AG2.13.2 and *A. rivipollensis* AG2.13.5 is the capacity to degrade D-sorbitol and D-serine which is negative for *A. rivipollensis.* This strain is also negative for gelatinases, pectinases, D-galacturonic, L-galactonic acid lactone, bromo-succinic acid, nalidixic acid, and lithium chloride but positive for sodium butyrate. On the other hand, the degradation of D-fucose is intermediate for *A. rivipollensis* but negative for *A. salmonicida* (Table 4). Differences in sugar and amino acid metabolism have been associated with niche-specific differentiation in *E. coli* pathotypes [91].

#### 3.2.2. Hemolytic Activity

**Figure 7:**
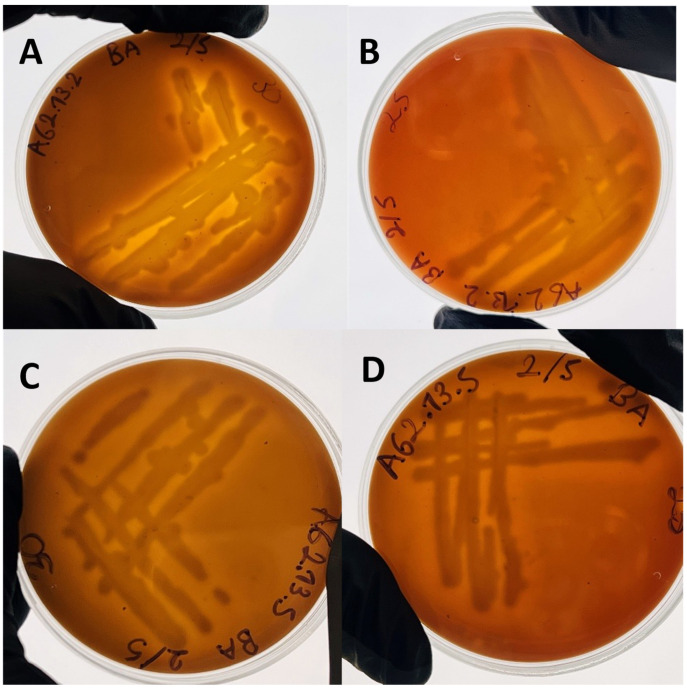
Hemolysis on seabream (*Sparus aurata*) blood agar of the strains *A. salmonicida* AG2.13.2 (**A**,**B**) and *A. rivipollensis* AG2.13.5 (**C**,**D**) at 25 °C (**right**) and 30 °C (**left**) after 24 h of incubation.

Very clear narrow zones of β-hemolysis were observed under the colony edges of the strains *A. salmonicida* AG2.13.2 and *A. rivipollensis* AG2.13.5 (Figure 7). According to the Bergey’s manual (2007), this characteristic is very common (>90%) for the *A. salmonicida*, *A hydrophila*, and *A. veronii* bv. *sobria* isolates. In particular, for the strain *A. salmonicida* AG2.13.2, hemolysis was clearly demonstrated when cultured at 30 °C (Figure 7A).

#### 3.2.3. Antibiotic Susceptibility

To classify the inhibition zone diameter (IZD), we used the quality control range established by Miller et al. (2003) for *A. salmonicida* [92] and the classifications established in the antibiotic susceptibility protocol for Gram-negative rods [34]. For the antibiotic flumequine, no cut-off values of the IZD could be obtained so we considered that an IZD equal or larger than 24 mm could be classified as sensitive (“S”).

The IZD of the strain *A. salmonicida* AG2.13.2 was smaller when compared to the reference strain of *A. salmonicida* subsp. *salmonicida* LMG3780 and *A. rivipollensis* AG2.13.5 for all the antibiotics tested, except for ampicillin for which the three strains showed some degree of resistance. AG2.13.2 was resistant to tetracycline, oxytetracycline, and oxolinic acid. This is very interesting, as none of the *A. veronii* bv. *sobria* strains from the incident in Agathonisi was resistant to oxytetracycline or tetracycline [4]. We also detected the presence of flumequine-resistant and oxolinic acid-resistant colonies after 48 h of incubation for the strain AG2.13.2. On the other hand, *A. rivipollensis* AG2.13.5 displayed colonies resistant to piperacillin after 24 and 48 h of incubation. The results of the antibiogram are shown in Table 5.

#### 3.2.4. In Vivo Virulence

**Figure 8:**
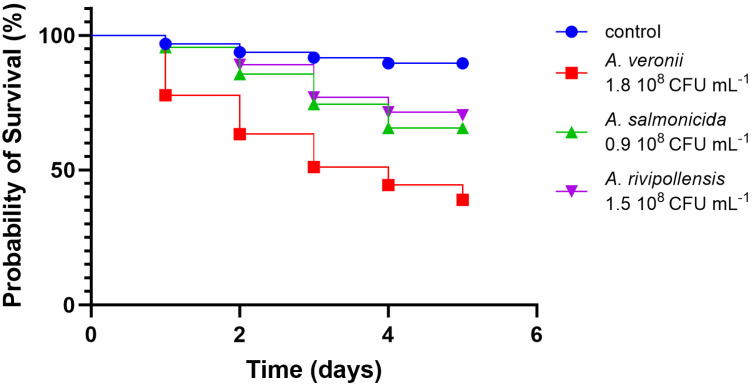
Survival curves plotted for the larvae challenged with different strains of *Aeromonas* strains. Log-rank (Mantel-Cox) analysis [χ^2^ (3, N = 367) = 60.99, *p* < 0.0001].

**Figure 9:**
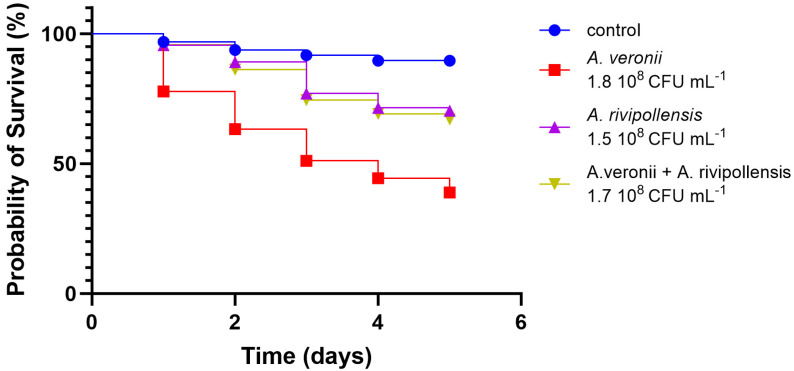
Survival curves plotted for the larvae challenged with *A. veronii* bv. *sobria* alone and in combination with *A. rivipollensis*. Log-rank (Mantel-Cox) analysis [χ^2^ (3, N = 370) = 61.67, *p* < 0.0001].

**Figure 10:**
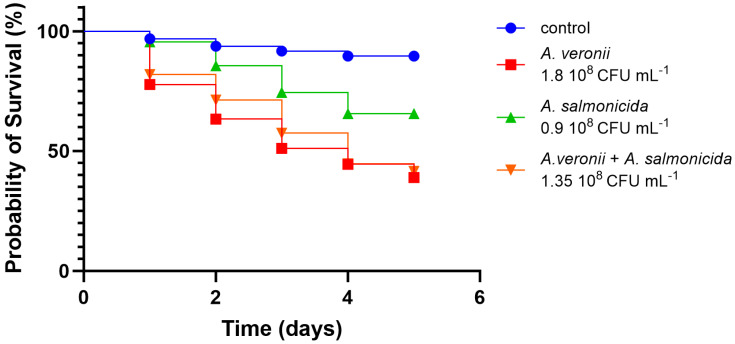
Survival curves plotted for the larvae challenged with *A. veronii* bv. *sobria* alone and in combination with *A. salmonicida*. Log-rank (Mantel-Cox) analysis [χ^2^ (3, N = 370) = 64.84, *p* < 0.0001].

Significant statistical variances emerged between the groups of larvae challenged with the bacterial suspensions alone and in pairs. The *A. veronii* bv. *sobria* strain that we used for the in vivo infection corresponds to the strain AG5.28.6 which was isolated in the same location of the co-isolates *A. salmonicida* AG2.13.2 and *A. rivipollensis* AG2.13.5. The larvae that were challenged with *A. veronii* bv. *sobria* AG5.28.6 had the lowest probability of survival in comparison to the larvae challenged with *A. salmonicida* AG2.13.2 and *A. rivipollensis* AG2.13.5 alone. For these two strains, the probability was greater than 50% in all tested groups (Figure 8). However, all the strains significantly reduced the probability of survival when compared with the untreated control. The larvae challenged with a combination of equal proportions of *A. veronii* bv. *sobria + A. rivipollensis* showed a significant increase in the probability of survival in comparison with the larvae challenged with *A. veronii* alone, suggesting that there is antagonism between the strains (Figure 9). When challenged with a combination of *A. veronii* bv. *sobria* + *A. salmonicida* in equal proportions, the probability of survival was as low as when they were challenged with *A. veronii* bv. *sobria* alone (Figure 10).

Antagonism between bacteria can be attributed to a diverse range of interactions. These interactions include competition for nutrients and other resources, the production of antibiotics and antimicrobial compounds, biofilm interactions, quorum sensing, contact-dependent inhibition, predation, and cross-feeding inhibition. Understanding these interactions is essential for understanding microbial communities and for the development of innovative strategies for the management of polymicrobial infections. Bacterial antagonism holds promise for applications in fields ranging from medicine to biotechnology and environmental science.

We know that bacteria interact with other microbes by producing effector molecules, often referred to as toxins. These proteins are released into the environment, where they engage with target cells possessing compatible receptors. These interactions can result in damage or even the death of the target cell. Such mechanisms are exemplified in substances like bacteriocins and fratricins [93]. Toxins can also be directly transmitted to neighboring bacteria through physical contact, a phenomenon known as contact-dependent interactions (CDIs). There is documented evidence of CDIs between *A. hydrophila* strains, as reported by Fernández-Bravo et al. (2019) [10]. In this case, a patient was infected with four *A. hydrophila* strains: three clonal (NF2, NF3, NF4) and one unique strain (NF1) containing a specific component of T6SSs, known as *tseC-tsiC*, an effector–immunity pair. Initially, the infection was misidentified as caused by a single strain, but metagenomics revealed it to be a multi-strain infection. Research has demonstrated that the T6SS and its effector–immunity pair, TseC-TsiC, were instrumental in the contact-dependent killing of the NF2 strain by the NF1 strain in the site of infection and resulted in the inability of NF2 to proliferate. CDIs are facilitated by two specialized secretion systems: type V (T5SS) and type VI (T6SS) [94]. The antibacterial functions of T6SSs are attributed to secreted antibacterial effector proteins and their corresponding immunity pairs (E-I pairs). These immunity proteins neutralize toxins in the secretor strains, preventing self-killing or harm to related bacterial cells. Thus, E-I pairs represent a novel toxin–antitoxin module that safeguards sister cells from toxic effectors. These E-I pairs have been identified in various bacterial species and can be categorized based on their targets: the cell wall, cellular membranes, and nucleic acids [93].

The available evidence strongly suggests that interactions among different microbes can significantly impact the outcomes of infections. This statement presents both challenges and interesting possibilities for infection treatment. However, our understanding of the dynamics of polymicrobial infections remains very limited. For our case, we need to consider that, in contrast to *A. veronii* AG5.28. 6 which harbors a T6SS cluster of genes, *A. rivipollensis* does not contain genes related to T5SSs or T6SSs and we also have no evidence of any proteins with antibacterial properties and therefore, the dynamics of this interaction are beyond the scope of this study.

## 4. Conclusions

*Aeromonas veronii* bv. *sobria* was the most virulent strain in the in vivo model and is recognized as the primary pathogen in European seabass Aeromoniasis. *A. salmonicida* and *A. rivipollensis* individually displayed minimal virulence towards seabream larvae. When *A. veronii* bv. *sobria* was combined with *A. rivipollensis*, the survival rate of larvae increased, suggesting a potential antagonistic interaction between the two strains, which requires further investigation.

*A. salmonicida* AG2.13.2, while less virulent, encodes significant virulence factors and antibiotic resistance genes, posing challenges for aquaculture, especially for non-salmonid species. *A. rivipollensis*, though not recognized as a fish pathogen, is able to acquire antibiotic resistance and virulence factors from the environment, raising concerns, particularly in the context of climate change [95]. *Aeromonas* species are known for their wide distribution and their ability to harbor and disseminate antibiotic resistance genes. Investigating the connection between these genes and insertion sequences (ISs) within *Aeromonas* could improve antibiotic resistance surveillance and provide new methods for studying antibiotic resistance [96].

## Figures and Tables

**Table 1:** General genome characteristics of the new *A. salmonicida* and *A. rivipollensis* strains compared with 2 reference strains retrieved from the NCBI.

	*A. salmonicida* AG2.13.2	*A. salmonicida* subsp. *Salmonicida* A449	*A. rivipollensis* AG2.13.5	*Aeromonas rivipollensis* KN-Mc-11N1
Length (bp)	4,798,364	4,702,402	4,464,750	4,508,901
Genes	4437	4813	4128	4186
CDS	4341	4673	4035	4025
Pseudogenes	48	238	59	140
rRNAs	1, 1, 1 (5S, 16S, 23S)	10, 9, 9 (5S, 16S, 23S)	1, 1 (16S, 23S)	11, 10, 10 (5S, 16S, 23S)
tRNA	88	108	84	124
ncRNAs	5	9	7	6

**Table 2:** Antibiotic resistance genes of the studied *Aeromonas* strains, *A. salmonicida* AG2.13.2, and A. *rivipollensis* AG2.13.5. Genes can be accessed through their locus tags or names.

Strain	Resistance Gene	Locus Tag	Resistance Profile
***A. salmonicida* AG2.13.2**	*cphA5*	LCG94_02875	Subclass B2 β-lactamase
	*tetE*, *tetR*	LCG94_08210, LCG94_08205	Tetracycline efflux pump
	*uhpT*	LCG94_00425	Fosfomycin
	*OXA-12*	LCG94_03955	Class D β-lactamase
	*ampC*	LCG94_12450	Class C beta-lactamase
	*tuf*	LCG94_09150	Elfamycin resistance gene
	*macB-tolC*	LCG94_11330, LCG94_00440	Macrolide efflux pump
	*qnrB*	LCG94_06945	Quinolone resistance pentapeptide
	*mdtL*	LCG94_10650, LCG94_03795	Multidrug efflux pump
	*uhpT*	LCG94_00425	Related to fosfomycin resistance
	*pgsA*	LCG94_15430	Protein-altering, cell wall charge-conferring antibiotic resistance
	*katG*	LCG94_17960	Isoniazid drug resistance
	*oxyR*	LCG94_12130	Hydrogen peroxide-inducible gene activator
***A. rivipollensis* AG2.13.5**	*macB-tolC*	RXZ09_11995, RXZ09_15840	Macrolide efflux pump
	*qnrB*	RXZ09_19125	Quinolone resistance pentapeptide
	*mdtL*	RXZ09_01745 RXZ09_18315	Multidrug efflux pump
	*ampC*	RXZ09_03805	Class C beta-lactamase
	*uhpT*	RXZ09_12110	Related to fosfomycin resistance
	*OXA-504*	RXZ09_18590	Class D β-lactamase
	*tuf*	RXZ09_17225	Elfamycin resistance gene
	*katG*	RXZ09_09250	Isoniazid drug resistance
	*oxyR*	RXZ09_03480	Hydrogen peroxide-inducible gene activator

**Table 3:** Characteristic peaks for 4 reference strains of different subspecies of *A. salmonicida* as reported by Fernández-Álvarez et al. (2016) [17]. For the reference strains, the peak mass values are shown as the arithmetic means of the m/z values of 4 replicates and for the strain AG2.13.2, there are no replicates.

*A. salmonicida* subsp. *salmonicida* ATCC33658	*A. salmonicida* subsp. *achromogenes* ATCC33659	*A. salmonicida* subsp. *masoucida* ATCC27013	*A. salmonicid*a subsp. *smithia* ATCC49393	*A. salmonicida* AG2.13.2
**2028.35**	2028.35	2028.35	2028.35	2027.3204
**2127.46**	**2105.56**	2127.46	**2461.93**	2128.6416
**3240.09**	2127.46	**3011.77**	**2920.31**	2524.7395
**3435.62**	**3112.82**	3240.09	**3082.85**	3042.0710
**3595.69**	**3293.22**	**3372.66**	3240.09	3151.8762
**4260.10**	**3400.97**	3435.62	**3435.62**	3597.1121
**4371.13**	3595.69	3595.69	**3739.54**	3842.3545
**4595.81**	4117.59	**3762.02**	4260.10	4171.2969
**5156.84**	**5105.56**	4260.10	4371.13	4258.7832
**5534.26**	6086.98	**4371.13**	4595.74	4347.0786
**6086.98**	6478.28	4595.81	5156.84	4468.8955
**6478.28**	**6804.55**	5156.84	5534.56	4592.4971
**6850.39**	6850.39	5534.26	**5930.05**	4655.8027
**7333.91**	7195.51	6086.76	6850.39	4700.5498
**8072.35**	7333.91	6477.70	**7283.06**	4976.9204
**8936.98**	8072.35	**6747.92**	8072.35	5051.1475
	**8884.05**	7195.51		5089.3784
	8936.98	7333.89		5156.0093
		8072.35		6086.2578
		8936.98		6306.0918
		**9014.04**		6481.3032
				7195.9482
				7333.4922
				7659.6675
				8343.4707
				8938.5283
				9185.4803
				9401.5156

The *A. salmonicida* subspecies-specific peaks are shown in bold in the reference strains columns.

**Table 4:** Results of the GEN III Microplate biochemical characterization of the strains of *A. salmonicida* and *A. rivipollensis*. Results are shown as positive (+), negative (−), or intermediate.

Substrate	*A. salmonicida* AG2.13.2	*A. rivipollensis* AG2.13.5	Substrate	*A. salmonicida* AG2.13.2	*A. rivipollensis* AG2.13.5
Negative control	−	−	Glycyl-L-proline	+	+
Dextrin	+	+	L-alanine	+	+
D-maltose	+	+	L-arginine	+	+
D-trehalose	+	+	L-aspartic acid	+	+
D-cellobiose	+	+	L-glutamic acid	+	+
Gentiobiose	−	−	L-histidine	+	+
Sucrose	+	+	L-pyroglutamic	−	−
D-turanose	−	−	L-serine	+	+
Stachyose	−	−	Lincomycin	+	+
Positive control	+	+	Guanidine HCL	+	+
pH 6	+	+	Niaproof 4	+	+
pH 5	−	−	Pectin	intermediate	−
D-raffinose	−	−	D-galacturonic	intermediate	−
α-d-lactose	−	−	L-galactonic acid lactone	intermediate	−
D-melibiose	−	−	D-gluconic acid	+	+
Β-Methyl-D-glucoside	+	+	D-glucuronic acid	−	−
D-salicin	−	−	Glucuronamide	intermediate	intermediate
N-acetyl-D-glucosamine	+	+	Mucic acid	−	−
N-acetyl-β-D-mannosamine	−	−	Quininic acid	−	−
N-acetyl-D-galactosamine	−	−	D-saccharic acid	−	−
N-acetyl neuraminic acid	−	−	Vancomycin	+	+
1% NaCl	+	+	Tetrazolium violet	+	+
4% NaCl	+	+	Tetrazolium blue	+	+
8% NaCl	−	−	P-Hydroxy-phenylacetic acid	−	−
A-D-glucose	+	+	Methyl pyruvate	+	+
D-mannose	+	+	D-lactic acid methyl ester	−	−
D-Fructose	+	+	L-lactic acid	−	−
D-galactose	+	+	Citric acid	+	−
3-methyl glucose	−	−	A-keto-glutaric acid	−	−
D-fucose	−	intermediate	D-malic acid	−	−
L-fucose	−	−			
L-rhamnose	−	−	L-malic acid	+	+
Inosine	+	+	Bromo-succinic acid	intermediate	−
1% sodium lactate	+	+	Nalidixic acid	+	−
Fusidic acid	−	−	Lithium chloride	+	−
D-Serine	+	−	Potassium tellurite	−	−
D-sorbitol	+	−	Tween 40	+	+
D-mannitol	+	+	γ-Amino-butyric acid	−	−
D-arabitol	intermediate	−	α- Hydroxy-butyric acid	−	−
Myo-inositol	−	−	β- Hydroxy-D, L-butyric acid	−	intermediate
Glycerol	+	+	A-keto-butyric acid	intermediate	−
D-glucose 6-PO_4_	+	+	Acetoacetic acid	+	+
D-Fructose 6-PO_4_	+	+	Propionic acid	intermediate	−
D-aspartic acid	−	−	Acetic acid	+	+
D-serine	+	+	Formic acid	+	+
Troleandomycin	+	+	Aztreonam	+	intermediate
Rifamycin SV	+	+	Sodium butyrate	intermediate	+
Minocycline	intermediate	−	Sodium bromates	−	−
Gelatin	+	−			

Differences between AG2.13.2 and AG2.13.5 are highlighted in grey, without considering intermediate reactions.

**Table 5:** Antibiotic susceptibility of the studied strains. The IZD of the different antibiotics were classified as sensitive (“S”), intermediate (“I”), or resistant (“R”) according to the protocol established by Hudzicki (2009) [34] and the quality control ranges of IZDs proposed by Miller et al. (2003) [92].

Strain	Drug	Content (μg)	IZD (mm) 24 h	Sensitivity
***A. salmonicida salmonicida* LMG3780**	Oxytetracycline	30	36	S
	Tetracycline	30	35	S
	Piperacillin	100	30	S
	Sulfamethoxazole	25	34.5	S
	Florfenicol	30	38	S
	Ampicillin	10	23	S
	Flumequine	30	38	S
	Oxolinic acid	2	35	S
***A. salmonicida* AG2.13.2**	Oxytetracycline	30	10	R
	Tetracycline	30	15	I
	Piperacillin	100	29.5	S
	Sulfamethoxazole	25	30	S
	Florfenicol	30	27	I
	Ampicillin	10	0	R
	Flumequine	30	24	S
	Oxolinic acid	2	11	R
***A. rivipollensis* AG2.13.5**	Oxytetracycline	30	33	S
	Tetracycline	30	34.5	S
	Piperacillin	100	34	S
	Sulfamethoxazole	25	32	S
	Florfenicol	30	30	S
	Ampicillin	10	0	R
	Flumequine	30	40	S
	Oxolinic acid	2	34	S

## Data Availability

All data are provided in the article.

## Supplementary Materials

The following supporting information can be downloaded at: https://www.mdpi.com/article/10.3390/pathogens12111337/s1, Figure S1: BUSCO statistics; Table S1: List of genomes downloaded from the NCBI; Table S2: Assembly statistics; Table S3: CRISPRcasFinder results; Table S4: PHASTER results; Table S5: Virulence factors details.
