# Supplementary material for: Polyinfection in Fish Aeromoniasis: A Study of Co-Isolated Aeromonas Species in Aeromonas veronii Outbreaks
Source: Pathogens. 2023 Nov 10;12(11):1337. doi: 10.3390/pathogens12111337 (PMC10674900; doi:10.3390/pathogens12111337)
Supplement: Supplementary file 1 [file pathogens-12-01337-s001.zip › Supplementary figure S1 and tables S1,S2,S3,S4-v2-Layout.pdf]

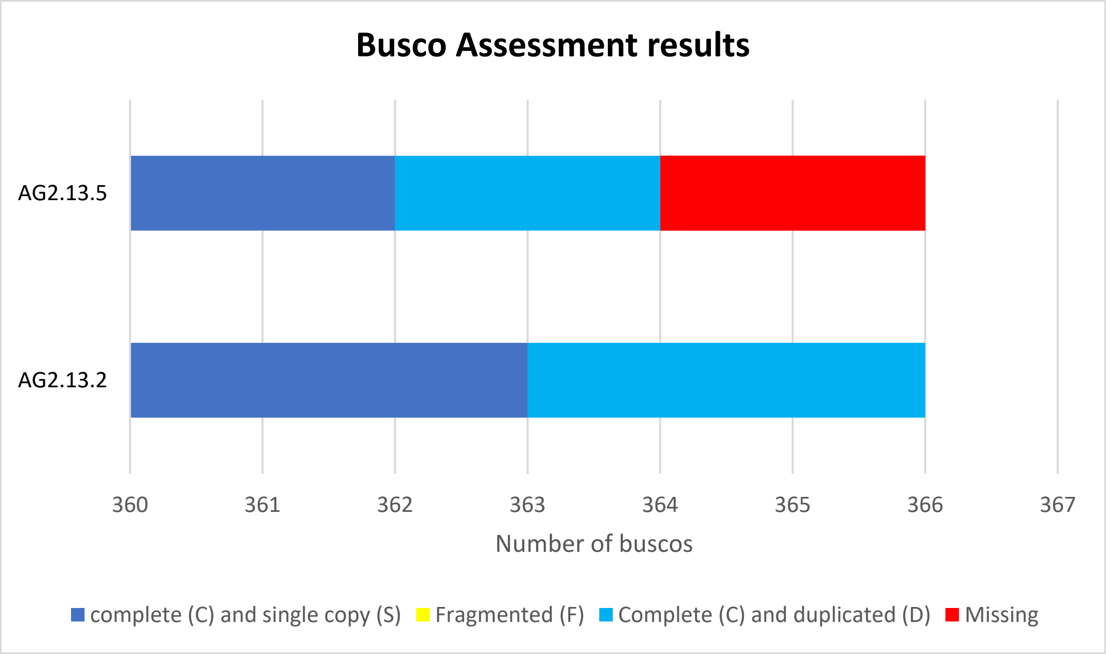


**Figure S1.** Busco Assessment results showing the number of copies of BUSCO genes complete (**C = 366**), complete and single copy (**S= 363**) complete and duplicated (D = 3), fragmented (**F=0**) and missing (**M =0**) (**n = 366**) in strain AG2.13.2 and complete (**C = 364**), complete and single copy (**S= 362**) complete and duplicated **(D = 2)**, fragmented (**F=0**) and missing (**M =2**) (**n = 366**) in strain AG2.13.5. .

***Table S1.*** List of genomes downloaded from the NCBI- Characteristics of the strains selected for genome similarity assessments and comparisons.

| Strain | GeneBank Assembly Accession number | Observations | Host/isolation source | Location | Date of isolation |
|---|---|---|---|---|---|
| *A. salmonicida A527* | GCA_002764135.1 | Mesophilic | *Macrobrachium rosenbergii* | Mumbai, India | May 2007 |
| *A. salmonicida* Y47 | GCA_001481535.2 | Mesophilic | *Gallus domesticus* | Mumbai, India | January 2006 to March 2008 |
| *A. salmonicida* Y567 | GCA_001466435.1 | Mesophilic | Fish | Mumbai, India | January 2006 to March 2008 |
| *A. salmonicida* Y577 | GCA_001481545.2 | Mesophilic | Fish | Mumbai, India | January 2006 to March 2008 |
| *A. salmonicida pectinolyitica 34mel* | GCA_000447435.2 | Mesophilic, pigment producer | Heavily polluted water, Matanza river | Buenos Aires, Argentina | 1988 |
| *A. salmonicida SWR-OG1* | GCA_012931585.1 | Mesophilic | *Epinephelus coioides* | Southern China Zhangzhou city, Fujian province | April 2018 |
| *A. salmonicida O23A* | GCA_002180335.1 | Dissimilatory arsenate reductase gene | Rock biofilm and bottom sediments | Zloty Stok gold mine in south-west Poland | 2010 |
| *A. salmonicida S121* | GCA_002214245.1 | Unavailable. Most likely belonging to *salmonicida* subspecies | *Salmo salar* | Yantai, China | August 2015 |
| *A. salmonicida masoucida RZ6S-1* | GCA_019443825.1 | Intermediate | *Scophthalmus maximus* | Yantai, China | December 2016 |
| *A. salmonicida salmonicida J409* | GCA_009858115.1 | Unavailable | *Anoplopoma fimbria* | British Columbia, Vancouver, Canadá | 2018 |

| | | | | | |
|---|---|---|---|---|---|
| *A. salmonicida salmonicida A449* | GCA_000196395.1 | Psychrophilic | *Salmo trutta* | Eure river, France | 1975 |
| *A. salmonicida salmonicida SHY16-3432* | GCA_008370735.1 | Psychrophilic | *Salvelinus fontinalis* | Quebec, Canadá | 2016 |
| *A. salmonicida AS1* | GCA_029153695.1 | Psychrophilic | Duck meat | Shandong, China | 2016 |
| *A. media T0.1-19* | GCA_013085485.1 | Misidentified | Sludge from bioreactor treating oxytetracycline bearing wastewater | Beijing, China | April 2016 |
| *A. media T5-1* | GCA_019455365.1 | Misidentified | Biofilm of synthetic bioreactor under oxytetracycline stress | Beijing, China | May 2017 |
| *A. media E31* | GCA_016653695.1 | | Water | Jinan, China | May 2018 |
| *A. media WS* | GCA_000287215.3 | High levels of melanin | Water | East Lake, Wuhan, Hubei, China | 2003 |
| *A. media R25-3* | GCA_013085765.1 | Synthetic bioreactors under oxytetracycline stress | Sludge from bioreactor treating oxytetracycline bearing wastewater | Beijing, China | October 2017 |
| *A. media R1-18* | GCA_013085725.1 | Synthetic bioreactors under oxytetracycline stress | Sludge from bioreactor treating oxytetracycline bearing wastewater | Beijing, China | May 2016 |
| *A. media R1-26* | GCA_014109865.1 | Synthetic bioreactors under oxytetracycline stress | Biofilm reactor | China | July 2018 |
| *Aeromonas rivipollensis KN-Mc-11N1* | GCA_003015165.1 | | *Myocastor coypus* | Nakdong River, South Korea | July 2017 |
| *Aeromonas rivipollensis G42* | GCA_010974915.1 | | Water | Johannesburg, South Africa | March 2018 |
| *Aeromonas veronii* bv *sobria* AG5.28.6 | GCA_003367135.1 | | *Dicentrarchus labrax* | East Aegean Sea, Greece | November 2015 |
| *Aeromonas veronii* bv *sobria* BIOO050A | GCA_004379215.1 | | *Dicentrarchus labrax* | East Aegean Sea, Greece | 2009 |
| *Aeromonas veronii* bv *sobria* NS6.15.2 | GCA_004379225.1 | | *Dicentrarchus labrax* | West Aegean Sea, Greece | November 2015 |
| *Aeromonas veronii* bv *sobria* NS | GCA_003367145.1 | | *Dicentrarchus labrax* | West Aegean Sea, Greece | 2009 |
| *Aeromonas veronii* bv *sobria* NS2 | GCA_004379205.1 | | *Dicentrarchus labrax* | West Aegean Sea, Greece | September 2015 |
| *Aeromonas veronii* bv *sobria* NS13 | GCA_008119755.1 | | *Dicentrarchus labrax* | West Aegean Sea, Greece | 2015 |
| *Aeromonas veronii* bv *sobria* NS22 | GCA_008119745.1 | | *Dicentrarchus labrax* | West Aegean Sea, Greece | September 2016 |
| *Aeromonas veronii* bv *sobria* PDB | GCA_003367105.1 | | *Dicentrarchus labrax* | West Aegean Sea, Greece | 2009 |
| *Aeromonas veronii* bv *sobria* VCK1 | GCA_003367095.1 | | *Dicentrarchus labrax* | East Aegean Sea, Greece | September 2015 |

***Table S2.*** Statistics of the genome-assembly report.

| Strain | Platforms | Assembler | Contigs | Largest Contig (bp) | N50 | GC% | coverage | Number of reads |
|---|---|---|---|---|---|---|---|---|
| ***A. salmonicida* AG2.13.2** | Illumina | Unicycler v0.4.8 | 65 | 402.057 | 168.750 | 58.74 | 256.397 | 6214779 |

| | | | | | | | | |
|---|---|---|---|---|---|---|---|---|
| ***A. rivipollensis* AG2.13.5** | Illumina | Unicycler v0.4.8 | 50 | 501.196 | 288.897 | 61.61 | 243.19 | 5502017 |

***Table S3.*** A. salmonicida AG2.13.2 CRISPR site prediction.

| CRISPR ID | Start | End | Length(bp) | Number of spacers | Direct repeat length(bp) |
|---|---|---|---|---|---|
| *CRISPR2* | 396098 | 396417 | 319 | 5 | 23 |

**Table S4.** *A. salmonicida* AG2.13.2 PHASTER prediction.

| PHASTER region | Start | End | Length(bp) | Number of Phage Hit Proteins |
|---|---|---|---|---|
| *INCOMPLETE PROPHAGE* | 4627372 | 4669096 | 41,700 pb | 41 |
